# The Effect of Language Concordance on Health Care Relationship Trust Score

**DOI:** 10.7759/cureus.39530

**Published:** 2023-05-26

**Authors:** Alexandria Daggett, Shaghayegh Abdollahi, Mehrtash Hashemzadeh

**Affiliations:** 1 Pediatrics, University of Arizona College of Medicine - Phoenix, Phoenix, USA; 2 Internal Medicine, Valleywise Health Medical Center, Phoenix, USA; 3 Biostatistics, University of Arizona College of Medicine - Phoenix, Phoenix, USA

**Keywords:** spanish language, language concordant physician, ad hoc interpreter, medical interpreter, health communication, trust in physician, health care relationship trust, patient-physician relationship, limited english proficiency, language concordance

## Abstract

Introduction: Various barriers exist for Limited English Proficient (LEP) patients in the United States healthcare system, including language barriers. To address language access, interpreters have been used as well as physicians who speak the same language (language concordance), with unknown effect. By studying the strength of patient-physician relationships under different communication parameters, such as the use of a variety of language services, we can better understand healthcare interactions and move toward optimizing patient care and health outcomes. This study investigates the importance of language-concordant care in LEP populations to build trusting patient-physician relationships.

Objective: To determine whether Spanish-speaking patients who receive health care from language-concordant (in this study, Spanish-speaking) physicians have higher total trust scores on the Health Care Relationship (HCR) Trust scale than patients who use professional or ad hoc interpreters.

Methods: This is a prospective survey conducted on Spanish-speaking adult patients coming to family and internal medicine outpatient clinics in the Phoenix, AZ, metro area. Of 214 recruited subjects, 176 completed the survey. Primary outcomes of the study: measured total mean Health Care Relationship (HCR) trust score among three groups (language concordant, professional interpreter, ad hoc interpreter). Secondary outcomes of the study: variance of trust scores among three groups for individual survey items.

Results: The group with language concordant providers had a mean trust score of 48.73, significantly higher than the mean for the group with ad hoc interpreters with a mean of 45.53 (p = 0.0090). Patients with a professional interpreter also had a higher mean trust score of 48.27 than the ad hoc interpreters (p = 0.0119). There were several individual questions where the professional language groups had statistically significantly higher HCR trust scores than the ad hoc interpreter group in terms of their trust in specific instances, i.e., involving the patient in discussing treatment options, making the patient feel worthy of the doctor’s time, and their doctor telling them the whole truth. There were no differences in overall mean scores or individual scores for the two professional language groups (language concordant providers and professional interpreters).

Conclusions: The results reinforce the current understanding that professionally acknowledged and trained speakers of a second language in the medical setting allow for the formation of stronger patient-physician relationships, specifically in terms of a patient’s trust in their physician. In addition to continuing to increase the availability of high-quality interpreters, the same effort should be placed on increasing the diversity of languages spoken by physicians to foster trusting patient-physician relationship formation.

## Introduction

As awareness of the importance of health equity increases, there is an increasing body of research regarding healthcare disparities. Barriers and health disparities are numerous in various patient populations, including patients with Limited English Proficiency (LEP). Patients with LEP have been shown to have increased difficulty understanding medical situations, medication labels, and instructions, and increased risk of adverse medication effects, likely due to miscommunication and subsequent misuse [1]. LEP patients also report lower utilization of health care services and fewer physician visits than patients with English fluency [2].

One method to address language barriers and poorer outcomes for LEP patients is the use of professional interpreter services. The use of professional interpreters has been shown to improve health outcomes for LEP patients and leads to increased time spent on patient education as compared to encounters without an interpreter [3]. Despite this data, interpreter use varies greatly [1]. Although federally funded clinics are legally required to provide interpreter services for LEP patients, significant disparities remain in the consistency of use, including the persistent use of ad hoc interpreters, i.e. patient family members and clinical staff without formal interpretation or language training. Even when used, interpreters do not resolve all communication barriers for LEP patients. Interpreter use has been shown to lead to no improvement and even worsening in the patient-reported perception of the care they received and interpersonal communication with their physician [3].

Language-concordant care, healthcare provided in the patient’s native language from a provider who fluently speaks the same language, may address both linguistic and social barriers for LEP patients. In a 2019 systematic literature review, researchers found that language concordance improves care for LEP patients in both subjective (e.g. patient satisfaction, empowerment to manage own health conditions) and objective (e.g. blood pressure control) measures [4]. In addition, interactions with language-concordant providers have demonstrated increased comprehension and overall health literacy when compared to interactions using professional interpreters [5]. These key differences in the success of communication may have effects on overall health outcomes and patient-physician relationship formation.

Effective healthcare communication requires that language barriers are addressed first and foremost. Good communication improves patient and physician satisfaction, reduces adverse events and errors, and overall improves patients’ physical and mental health outcomes [6]. This communication helps to build a strong therapeutic relationship. Patient satisfaction with the patient-physician relationship has been shown to be highest with patients who saw language-concordant providers, followed by those who used a professional interpreter [7]. The lowest satisfaction was for patients who did not use an interpreter but believed one should have been called [7]. Language-discordant patients were approximately 60% more likely to rate the interpersonal care during their encounter as fair or poor as compared to language-concordant patients, whether or not an interpreter was used [3].

In addition to clear communication, trust is essential to the patient-physician relationship. Patients must believe in the good character of their physician and trust their knowledge and integrity if they are to allow a certain degree of physician autonomy in helping determine a care plan [8]. Once built, a foundation of trust supports continued success in communication and relationship maintenance. High levels of patient trust in their physician have been associated with high scores for physical, psychological, social, and environmental health-related quality of life [9], and various health outcomes [10,11]. Patient trust in their physician has been associated with improved self-management of chronic illness [12,13], improved treatment and medication adherence [13-16], and increased rates of preventive health tasks [17]. Conversely, measures of distrust in healthcare providers have been associated with poorer health-related quality of life and increased anxiety in seeking necessary treatment [18].

It is well-established that trust, clear communication, strong patient-physician relationships, and elimination of language barriers are independently important in healthcare. It has not yet been characterized how these factors interplay with each other for LEP patients and how language-concordant care may play a role in improving outcomes. This study investigates the importance of language-concordant care in LEP populations as a means of building trusting patient-physician relationships. Language concordance in Spanish was selected due to the high prevalence of Spanish-speaking patients and physicians in the United States, particularly in the Southwest.

We hypothesized that patients whose physicians communicated with them in their native Spanish would report higher trust scores than those who communicated indirectly via a professional interpreter or ad hoc interpreter.

## Materials and methods

This was a prospective study conducted via survey. Participants were recruited from three Phoenix-area internal and family medicine health centers during in-person and telemedicine appointments and selected based on their preferred language of Spanish.

Participants were included if they spoke: Spanish as their native language, Spanish as their preferred primary language; Spanish as their preferred language for health interactions; some English but used Spanish during their appointment. All participants were 18 years or older and saw a physician at their appointment. Participants were excluded from the study if they: were bilingual in Spanish/English and completed their encounter in English; communicated with the physician primarily in English with some Spanish use; spoke Spanish but completed the encounter in another language (e.g., a native or indigenous language with the use of an interpreter).

Eligible participants were invited to participate during the rooming process and completed their scheduled appointment as usual. They completed the survey following their encounter with their physician. Of 214 participants recruited, 176 surveys were completed in their entirety and used in the analysis (response rate of 82.2%). Patients had the choice to complete the survey independently or have the survey read to them by a research assistant in the case of a telemedicine appointment or limited literacy. Incomplete surveys included those without adequate information filled in to sort them into appropriate study groups or with missing answers.

The primary study outcome was the mean total Health Care Relationship Trust score reported by each group. Secondary outcomes were the mean scores for individual survey items in regard to different facets of trust in a patient-physician relationship. The surveys were grouped into three study groups based on the format of language used during the appointment. Group A included appointments where the Spanish-speaking physician spoke directly with their patient in Spanish (language-concordant interaction). Group B included interactions where ad hoc interpreters were used, defined as patient family members, clinical staff without formal interpretation or language training and certification, or any different language assistance other than a professional interpreter. Group C included appointments where a professionally trained Spanish interpreter was used. Language concordance included Spanish-speaking interactions only for this study design. Providers who would normally speak Spanish with their patients with permission and linguistic approval from their respective hiring institutions continued to speak Spanish throughout this study. There were no additional proficiency tests or language assessments completed for this study.

Health care relationship (HCR) trust scale

The survey used was the Health Care Relationship (HCR) Trust Scale, a Likert scale to measure trust in a patient-physician relationship [19]. This scale was previously tested for internal consistency, test-retest reliability, and construct validity [19], and subsequently adapted to a Spanish version [20]. The survey was scored from zero to four for each of 13 questions and summed for a total score.

Statistical methods

The Kruskal-Wallis test was used to analyze patient answers in the three study groups. If a significant difference existed (p < 0.05), we used the Dwass, Steel, Critchlow-Fligner Method for multiple comparisons to compare three categories for each question. The Kruskal-Wallis tests were performed using SAS version 9.4 (SAS Institute Inc., Cary, NC).

## Results

When comparing total HCR Trust score, both groups with professional Spanish services (A and C) had a statistically significant higher total HCR Trust score than the ad hoc interpreter group (B); Spanish-speaking physician encounters (mean 48.73) scored greater than ad hoc interpreter encounters (mean 45.53), p = 0.0090; Professional interpreter encounters (mean 48.27) scored greater than ad hoc interpreter encounters, p = 0.0119. There was no significant difference between the two professional language groups (Table 1).

**Table 1:** Results of the Kruskal-Wallis test comparing scores for language-concordant encounters (A, n=67), ad hoc interpreter encounters (B, n=17) and professional interpreter encounters (C, n=92). Asterisks show a significant pairwise difference (p < 0.05).

Questions	Groups	Mean	Comparisons Dwass, Steel, Critchlow-Fligner Method	p-value
1. How often does your doctor discuss options and choices with you before health care decisions are made?	Language concordant physician (A)	3.78	A vs. B	0.0232*
	Ad hoc interpreter (B)	3.41	A vs. C	0.6879
	Professional interpreter (C)	3.60	B vs. C	0.1145
2. My doctor is committed to providing the best care possible.	Language concordant physician (A)	3.88	A vs. B	0.0371*
	Ad hoc interpreter (B)	3.65	A vs. C	0.9451
	Professional interpreter (C)	3.85	B vs. C	0.0117*
3. My doctor is sincerely interested in me as a person.	Language concordant physician (A)	3.88	A vs. B	0.0567
	Ad hoc interpreter (B)	3.65	A vs. C	0.9580
	Professional interpreter (C)	3.82	B vs. C	0.0824
4. My doctor is an excellent listener.	Language concordant physician (A)	3.91	A vs. B	0.0679
	Ad hoc interpreter (B)	3.71	A vs. C	0.5600
	Professional interpreter (C)	3.82	B vs. C	0.2984
5. My doctor accepts me for who I am.	Language concordant physician (A)	3.90	A vs. B	0.1162
	Ad hoc interpreter (B)	3.71	A vs. C	0.8407
	Professional interpreter (C)	3.82	B vs. C	0.2465
6. My doctor tells me the complete truth about my health-related problems.	Language concordant physician (A)	3.88	A vs. B	0.1804
	Ad hoc interpreter (B)	3.71	A vs. C	0.4959
	Professional interpreter (C)	3.89	B vs. C	0.0149*
7. My doctor treats me as an individual.	Language concordant physician (A)	3.79	A vs. B	0.2143
	Ad hoc interpreter (B)	3.71	A vs. C	0.9030
	Professional interpreter (C)	3.73	B vs. C	0.3488
8. My doctor makes me feel that I am worthy of his/her time and effort.	Language concordant physician (A)	3.91	A vs. B	0.0162*
	Ad hoc interpreter (B)	3.65	A vs. C	0.8471
	Professional interpreter (C)	3.90	B vs. C	0.0018*
9. My doctor takes the time to listen to me during each appointment	Language concordant physician (A)	3.93	A vs. B	0.0350*
	Ad hoc interpreter (B)	3.71	A vs. C	0.9465
	Professional interpreter (C)	3.87	B vs. C	0.0513
10. I feel comfortable talking to my doctor about my personal issues.	Language concordant physician (A)	3.82	A vs. B	0.0310*
	Ad hoc interpreter (B)	3.59	A vs. C	0.4893
	Professional interpreter (C)	3.87	B vs. C	0.0008*
11. I feel better after seeing my doctor.	Language concordant physician (A)	3.88	A vs. B	0.0144*
	Ad hoc interpreter (B)	3.59	A vs. C	0.8116
	Professional interpreter (C)	3.87	B vs. C	0.0016*
12. How often do you think about changing to a new doctor?	Language concordant physician (A)	1.55	A vs. B	0.4518
	Ad hoc interpreter (B)	2.29	A vs. C	0.9574
	Professional interpreter (C)	1.52	B vs. C	0.3899
13. How often does your doctor consider your need for privacy?	Language concordant physician (A)	3.73	A vs. B	0.1692
	Ad hoc interpreter (B)	3.59	A vs. C	0.4941
	Professional interpreter (C)	3.77	B vs. C	0.0140*
Total HCR Trust Score	Language concordant physician (A)	48.73	A vs. B	0.0090*
	Ad hoc interpreter (B)	45.53	A vs. C	0.9975
	Professional interpreter (C)	48.27	B vs. C	0.0119*

Participants with a language concordant physician (A) scored statistically significantly higher mean scores for six of the 13 individual questions when compared to ad hoc interpreter (B). Participants rated higher scores for how often their doctor discussed options and choices with them before health care decisions were made (A mean = 3.78; B mean = 3.41; p = 0.0232), their doctor committing to providing the best care possible (A mean = 3.88; B mean = 3.65; p = 0.03710), their doctor making the participant feel worthy of his/her time and effort (A mean = 3.91; B mean = 3.65; p = 0.0162), their doctor taking time to listen to them during each appointment (A mean = 3.93, B mean = 3.71; p = .0.0350), feeling comfortable talking to their doctor about personal issues (A mean = 3.82, B mean 3.59; p = 0.0310), and feeling better after seeing their doctor (A mean = 3.88, B mean 3.59; p = 0.0144).

Participants who used a professional interpreter (C) had statistically significantly higher mean scores for six of the 13 survey questions when compared to ad hoc interpreter use (B). Participants rated higher scores for their doctor committing to providing the best care possible (C mean = 3.85, B mean = 3.65; p = 0.0117), their doctor telling the complete truth about health-related problems (C mean = 3.89, B mean = 3.71; p = 0.0149), their doctor making them feel worthy of his/her time and effort (C mean = 3.90, B mean = 3.65; p = 0.0018), feeling comfortable talking to their doctor about personal matters (C mean = 3.87, B mean = 3.59; p = 0.000800), feeling better after seeing their doctor (C mean = 3.87, B mean = 3.59; p = 0.00160), and their doctor considering their need for privacy (C mean = 3.77, B mean = 3.59; p = 0.0140). Mean total and individual question scores were similar between the groups whose encounters had professional language services (A and C) without any statistically significant difference.

## Discussion

Our hypothesis that participants whose physicians communicated with them in their native Spanish language would report higher trust scores than those whose physicians communicate indirectly via professional or ad hoc interpreters was not fully supported. The data did not demonstrate any significant difference between the HCR trust scores of language-concordant physicians and professional interpreters. There were, however, significant differences between both professional Spanish groups and the ad hoc interpreters. These results reinforce the current understanding that professionally acknowledged and trained speakers of a second language in the medical setting allow for the formation of a better patient-physician relationship, especially in terms of patient trust in their physician.

Various limitations to this study were identified. This study is the first of its kind analyzing how trust and patient-physician relationships may be affected by language. The use of multiple research assistants may have led to variance in survey instructions which could partially explain the 17.7% of surveys that were incomplete or incorrectly filled out, this could also be attributed to the lack of appropriate literacy screening for participants. No data was collected on the physician participants’ level of Spanish fluency when choosing to speak directly with patients. In future administrations of this survey, it may be helpful to require physicians to complete a formal Spanish evaluation to determine if they truly have the fluency to conduct an interaction fully in Spanish and if their level of proficiency had any additional effects on the participants’ HCR trust score. There are known limitations of Likert scales to create objective measurements of subjective experiences, which certainly contribute to the findings in this study. This leads the researchers to continue to wonder if there is a difference in the trust formed between patient and physician that may have been studied or quantified differently. Additionally, this study was conducted during the global coronavirus disease 2019 (COVID-19) pandemic which caused international concern and notable changes in trust in the healthcare system. Though its potential impact is immeasurable, we wonder if the setting of the pandemic had any effects on trust scores received and affected the outcomes between groups.

Future research is needed to continue exploring the effects of language on the quality of healthcare encounters and on patient-physician relationship formation. It would be beneficial to explore the effect of language concordance on factors other than trust, such as knowledge, regard, and loyalty. It is important to explore the effects of languages other than Spanish on relationship formations as these relationships are likely multifactorial and may be affected by components such as culture, socioeconomic status, and linguistics. It is also interesting to consider whether a statistically significant difference in trust, specifically, has a clinically significant impact on patient adherence to treatment plans, returning for follow-up with the same doctor, and other markers of a good therapeutic relationship, which was outside the scope of this study.

## Conclusions

Clinically, it is important to bear in mind the importance of meeting patients’ basic needs, one of which is language, in order to provide health care. Speaking Spanish directly to patients and using professionally trained Spanish interpreters is likely to improve the formation of trust in the patient-physician relationship when compared to non-professional language assistance. It is necessary to provide adequate language services for LEP patients and their families, and important to avoid the use of ad hoc interpreters whenever possible. As patient populations in the US continue to grow and diversify, it is essential that healthcare systems continue increasing the diversity of their workforce. Doing such will increase opportunities for improved access to language-concordant care and the possibility of improved trust in patient-physician relationships for LEP patients.

## Appendices

**Table 2:** HCR Trust Score in English HCR: Health care relationship

	0 = none of the time	1 = some or little of the time	2 = occasionally or a moderate amount of the time	3 = most of the time	4 = all of the time
How often does your doctor discuss options and choices with you before health care decisions are made?					
My doctor is committed to providing the best care possible.					
My doctor is sincerely interested in me as a person.					
My doctor is an excellent listener.					
My doctor accepts me for who I am.					
My doctor tells me the complete truth about my health-related problems.					
My doctor treats me as an individual.					
My doctor makes me feel that I am worthy of his/her time and effort.					
My doctor takes the time to listen to me during each appointment.					
I feel comfortable talking to my doctor about my personal issues.					
I feel better after seeing my doctor.					
How often do you think about changing to a new doctor?					
How often does your doctor consider your need for privacy?					

**Table 3:** HCR Trust Score in Spanish HCR: Health care relationship

	0 = ninguno de los casos	1 = una parte o pocas veces	2 = de vez en cuando	3 = la mayor parte del tiempo	4 = todo el tiempo
¿Con qué frecuencia su doctor discute sobre las opciones y elecciones con usted antes de tomar decisiones de atención de salud?					
Mi doctor se encuentra comprometido a ofrecer la mejor atención posible					
Mi doctor se encuentra sinceramente interesado en mí como persona					
Mi doctor es una persona que escucha excelentemente					
Mi doctor me acepta por quién soy					
Mi doctor me dice totalmente la verdad acerca de mis problemas de salud					
Mi doctor me trata como un individuo					
Mi doctor me hace sentir que soy digno de su tiempo y esfuerzo					
Mi doctor dedica su tiempo a escucharme durante cada cita					
Me siento cómodo hablando con mi doctor sobre cuestiones personales					
Me siento mejor después de ver a mi doctor					
¿Con qué frecuencia piensa en cambiar de doctor?					
¿Con qué frecuencia su doctor considera su necesidad de privacidad?					
